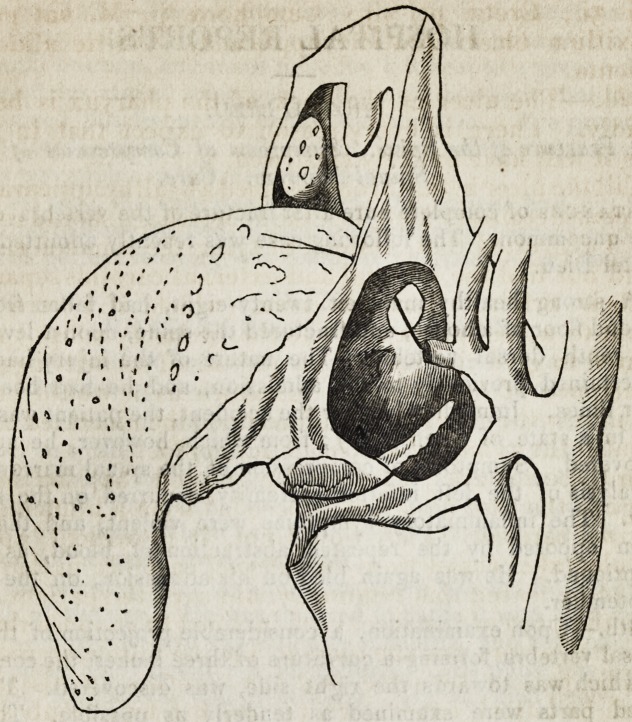# Hemorrhage from an Ulcer in the Fauces; Ligature of the Common Carotid

**Published:** 1829-12

**Authors:** Herbert Mayo

**Affiliations:** 19, George Street, Hanover Square


### ULCER IN THE FAUCES.

### *Hemorrhage from an Ulcer in the Fauces; Ligature of the common Carotid.*

*To the Editors of the London Medical and Physical Journal.*

GENTLEMEN: If the following case appear to you of sufficient interest, you will oblige me by giving it a place in your Journal.

John Webb, ætat. twenty-three, was admitted into the Middlesex hospital, on the evening of the 18th of October: those who brought him stated that he had suddenly lost a considerable quantity of blood from an ulcer in the fauces; but the hemorrhage had now stopped, and no apprehension was entertained of its immediate return.

The next morning, however, towards nine o'clock, the bleeding burst out afresh, and in a few minutes two quarts of blood were lost. The house-surgeon, Mr. LAIDLAW, hastened to the ward, and with great promptness compressed the carotid artery on the affected side, when the hemorrhage ceased. Notice being sent to me of what had happened, I went instantly to the hospital, and found the patient faint to the last degree, pale and bloodless.

On examining the fauces, I saw a ragged clot of blood adhering to the right side of the pharynx, while the left tonsil and adjacent surface appeared clear and healthy. I proceeded, therefore, without loss of time, to tie the right common carotid in the middle of the neck. Scarcely a drop of blood flowed from the incision made for this purpose; the pulse in the artery, when it was exposed, was exceedingly feeble; the internal jugular vein lay shrunk and collapsed.

After the operation the patient several times fell into an alarming state of faintness; but having taken some brandy, some Spiritus Ammoniæ Aromaticus in water, and some strong and spiced broth, he gradually rallied.

A few minutes after the artery was tied, I inquired of this patient whether he saw equally well with both eyes. He closed his eyes alternately, to ascertain the fact; and remarked, that his vision with the right eye was dim and obscure, while he saw distinctly with the left. At this time I could perceive no pulse in the right temporal artery. During the afternoon, distinctness of vision with the right eye returned; at the same time the pulsation of the right facial artery could be felt, though it was much less forcible than that of the left. The patient stated that he felt considerable throbbing in the left side of the head. He dozed much during the day, and slept well the following night.

The next morning an attempt was made to learn the history of his indisposition. The account which he then gave, however, and has since repeated, is very imperfect. He states that, for the last four months, he has had a sore throat; that, three months ago, spots broke out upon his chest and legs; that he took pills for these complaints during the space of three weeks, and that his mouth was affected about a fortnight; that, under this treatment, the spots upon his chest went away; that, for the last six weeks, he has taken no medicine, but has used a gargle; and that his throat he has latterly conceived to be getting better. The spots on the legs and thighs have left superficial ulcers, (which, when they were shown for the first time some days after his admission, were healing.) He states that he had a gonorrhœa a year ago; sores on the private parts never.

The appearance of the throat on the 20th was the following: The right margin of the uvula, and the edge of the right side of the soft palate, were in a state of ulceration; the right tonsil was entirely destroyed, together with the posterior arch of the palate; the right side, and the greater part of the posterior surface of the pharynx, were ulcerated, and covered with viscid puriform secretion; at one part a portion of ashy slough adhered to the surface.

℞. Decoct. Cinch. cum Acid. Sulph. dilut. ♏ viij. ter die.—Gargarisma e Chloridis Calcis gr. xv. cum Aquâ distillatâ ℔i.

The patient's strength improved daily; but the appearance of the throat was not materially changed before the 31st, when, in place of using a gargle, he was directed to fumigate the fauces with the vapour of a scruple of cinnabar. The following day, the whole of the ulcerated surface was covered with florid granulations.

On the 3d of November, the fifteenth day after the operation, the ligature came away from the artery. The ulcer of the pharynx had began to cicatrise.—P. in usu medicam. with full diet and a pint of porter daily.

On the 16th, the right side of the pharynx and palate had cicatrised. The ulcerated surface which remained was situated at the middle of the back part of the pharynx: it was an inch in length, half an inch in breadth; the ulcer yellow, and at one part deeply excavated; the cicatrised surface around of a bright red.—℞. Pil. Hydrarg. gr. vij. cum Extract. Hyoscyami gr. iij. o. n.—℞. Hydrarg. præcip.

albi ʒi.; Cretæ pt. ʒij.; Camphoræ ʒi. M. fiat pulvis; pauxillum ulceri quotidie applicand. Omitte alia medicamenta.

22d.—The ulcer at the back of the pharynx is healing rapidly. There is every reason to expect that in a few days it will have entirely cicatrised.

Till the ulcer in the throat assumed a healthy appearance, I felt considerable anxiety lest the hemorrhage should return; and I regretted that, instead of tying the common carotid, I had not tied the internal and external carotids separately at their origin. This operation (which, if I were again called to a similar case, I should probably adopt,) is calculated to give the patient an additional security against a recurrence of hemorrhage; inasmuch as it would cut off, not merely the *direct flow* of blood upon the ulcerated artery, but also the *principal anastomostic supply*.

In these remarks, I take it for granted that the hemorrhage in the case described proceeded from a branch of the *external* carotid. They would not, it is evident, apply if the ulcerated vessel were the *internal* carotid: in that case, it is to be feared that nothing would save the patient.

In the present instance, I am inclined to suppose that the ulcerated artery was the *lingual*, for the following reasons: 1. The patient tells me that the soreness in his throat, which he latterly experienced, seemed to him deeply seated within the angle of the jaw; and thus referred to the exact situation of the lingual artery, which, as regards the cavity of the fauces, is singularly exposed and superficial at this part. 2. In a case attended by Dr. Watson, in some respects very parallel to Webb's, where the patient died through hemorrhage into the fauces from ulceration, the artery which bled was proved, by dissection, to be the lingual artery.

This case is described in the third volume of the Medical Gazette, page 157. The immediate cause of death was suffocation: the blood, which, in his faint state, the patient could not spit out of his fauces, flowed into the windpipe. The carotid artery being afterwards injected, I made a careful examination of the parts, which are preserved in the museum in Great Windmill street: the adjoined sketch was taken from the preparation. The shaded surface in the figure represents the cavity of an abscess, in which the cornu of the os hyoïdes lay denuded and carious, and into which the lingual artery, that had been divided by ulceration, opened. The abscess communicated with the fauces through an oval aperture immediately below the tonsil.

Cases similar to the preceding are of rare occurrence. In some the first hemorrhage is fatal; in others, the ulcerated artery, having bled for a time, spontaneously closes, and the bleeding does not recur: in others, the patient is carried off by a return of hemorrhage. In the case of Webb, there can be little doubt that the latter result would have ensued if the artery had not been tied. I believe the case to be the first of the kind in which this operation has been performed: it is extremely gratifying to me to have to state that the practice has proved successful.

I remain, gentlemen,

Your obedient servant,

HERBERT MAYO.

19, *George street, Hanover square;*
*November 22d,* 1829.

## Figures and Tables

**Figure f1:**